# Pain sensitivity at rest and during muscle contraction in persons with rheumatoid arthritis: a substudy within the Physical Activity in Rheumatoid Arthritis 2010 study

**DOI:** 10.1186/s13075-018-1513-3

**Published:** 2018-03-15

**Authors:** Monika Löfgren, Christina H. Opava, Ingrid Demmelmaier, Cecilia Fridén, Ingrid E. Lundberg, Birgitta Nordgren, Eva Kosek

**Affiliations:** 1 Department of Clinical Sciences, Danderyd Hospital, Karolinska Institutet, S-182 88 Stockholm, Sweden; 2 Department of Rehabilitation Medicine, Danderyd Hospital, Building 60, S-182 88 Stockholm, Sweden; 3 Division of Physiotherapy, Department of Neurobiology, Care Sciences and Society, Karolinska Institutet, Huddinge, Sweden; 4 Rheumatology Clinic, Karolinska University Hospital, Solna, Sweden; 5 Rheumatology Unit, Department of Medicine, Karolinska University Hospital, Solna, Karolinska Institutet, Stockholm, Sweden; 6 Functional Area Occupational Therapy & Physiotherapy, Allied Health Professionals’ Function, Karolinska University Hospital, Stockholm, Sweden; 7 Department of Clinical Neuroscience, Karolinska Institutet, Stockholm, Sweden; 8 Department of Neuroradiology, Karolinska University Hospital, Stockholm, Sweden; 9 Stockholm Spine Centre, Stockholm, Sweden

**Keywords:** Arthritis, Exercise, Pain measurement, Pain threshold

## Abstract

**Background:**

We aimed to explore pressure pain sensitivity and the function of segmental and plurisegmental exercise-induced hypoalgesia (EIH) in persons with rheumatoid arthritis (RA) compared with healthy control subjects (HC).

**Methods:**

Forty-six participants with RA (43 female, 3 male) and 20 HC (16 female, 4 male) participated in the study. Pressure pain thresholds, suprathreshold pressure pain at rest, and segmental and plurisegmental EIH during standardised submaximal contractions were assessed by algometry. Assessments of EIH were made by performing algometry alternately at the contracting (30% of the individual maximum) right *m. quadriceps* and the resting left *m. deltoideus*.

**Results:**

Participants with RA had higher sensitivity to pressure pain (RA, 318 kPa; HC, 487 kPa; *p* < 0.001), suprathreshold pressure pain 4/10 (RA, 433 kPa; HC, 638 kPa; *p* = 0.001) and suprathreshold pressure pain 7/10 (RA, 620 kPa; HC, 851 kPa; *p* = 0.002) than HC. Segmental EIH (RA, 0.99 vs 1.27; *p* < 0.001; HC, 0.89 vs 1.10; *p* = 0.016) and plurisegmental EIH (RA, 0.95 vs 1.36; *p* < 0.001; HC, 0.87 vs 1.31; *p* < 0.001) increased significantly during static muscle contraction in both groups alike (*p* > 0.05).

**Conclusions:**

Our results indicate a generally increased pain sensitivity but normal function of EIH among persons with RA and offer one possible explanation for pain reduction observed in this group of patients following clinical exercise programmes.

**Trial registration:**

ISRCTN registry, ISRCTN25539102. Retrospectively registered on 4 March 2011.

## Significance and innovations

- Persons with rheumatoid arthritis (RA) have a generally increased pressure pain sensitivity.
- Static muscle contraction reduces pressure pain among both persons with RA and control subjects with no major pain alike.
- Our results offer one possible explanation for pain reduction observed in patients with RA following clinical exercise programmes.

## Background

Pain, tenderness and impaired muscle function are common in persons with rheumatoid arthritis (RA) [1]. Pain is the most common and disabling symptom [2] and is also found to be the main predictor of general health perception in patients with RA [3]. Pain is assumed to be linked to inflammation, but many persons with RA have continued pain despite adequately controlled inflammation [2]. The mechanisms behind non-inflammatory pain in RA, commonly considered as multifactorial, are not fully understood. In RA it might be caused by peripheral mechanisms, such as peripheral joint damage [4], possibly inducing peripheral sensitisation. Central mechanisms might also contribute to pain in RA by amplifying ascending nociceptive signalling due to sensitisation in ascending nociceptive pathways, increased descending facilitation and/or impaired descending inhibitory pathways (i.e., central sensitisation) [4, 5]. Previous findings support the presence of central pain mechanisms in RA. Thus, lower pain thresholds were found in patients with RA compared with healthy control subjects (HC), both at joints and at non-inflamed tissue, in studies using pressure algometry [5, 6]. Furthermore, hypersensitivity to thermal stimuli has been reported at both non-articular and articular sites [7]. Also, a general increase in pain sensitivity was found in patients with long-standing RA (>5 years) compared with those with more recent onset (<1 year) [5]. The data indicated that the generalised alteration of pain processing was due to sensitisation of central nociceptive neurons and/or increased activity in the descending facilitatory pathways, because normal function of conditioning pain modulation was found [5].

Data derived from animal studies indicate an interaction of opioid and serotonergic mechanisms to promote exercise-induced hypoalgesia (EIH) [8]. Also, endogenous opioids have been implicated during EIH in humans, but mainly during high-intensity and long-duration exercise [8, 9]. Recently, a genetic association study in humans showed that genetically inferred increased opioid signalling in combination with decreased serotonergic signalling produced better EIH in HC as well as in patients with fibromyalgia (FM). The study revealed antagonistic interactions between opioid and serotonergic signalling upon EIH [10]. On a behaviour level, exercise activates pain-inhibitory mechanisms in healthy persons with subsequent increase of pain thresholds during muscle contraction [11]. Persons with central sensitisation who were diagnosed with, for example, FM or FM with comorbid chronic fatigue syndrome respond differently [9, 10, 12–14], owing to a dysfunction of endogenous pain-inhibitory mechanisms during exercise (i.e., dysfunctional EIH). In patients with osteoarthritis (OA) in the hip or knee, we found increased pain sensitivity but normal function of EIH during muscle contractions [15]. These results align with the results of our previous pilot study where pain sensitivity was explored during standardised submaximal static contractions in a small sample of ten postmenopausal women with RA and indicated decreased pain thresholds compared with those in HC, but no dysfunction of segmental or plurisegmental EIH [16]. If these results can be replicated in larger samples including both men and women of different ages, they may offer one explanation for pain reduction following exercise [17]. The aim of the present study was thus to explore pressure pain sensitivity and the function of segmental and plurisegmental EIH in persons with RA compared with HC.

## Methods

### Participants

Forty-six persons (43 women, 3 men; mean age, 61 years [SD, 9]) with RA according to the American College of Rheumatology criteria [18], with a median disease duration of 9 years and a median activity limitation of 0.5 according to the Health Assessment Questionnaire Disability Index (HAQ-DI), participated in the present study. They represented those with complete data among the 70 participants in the Physical Activity in Rheumatoid Arthritis (PARA) 2010 study [19] volunteering to participate in the assessments of pressure pain thresholds (PPTs) and EIH. There was no difference between those with complete and incomplete data in important background data or baseline data, such as disease duration, pain scores and PPT levels.

The participants in the PARA 2010 study were recruited from the Swedish Rheumatology Quality Registers for an intervention study with a health-enhancing physical activity (HEPA) support programme, for which they were eligible if aged 18–75 years, independent in daily living (HAQ-DI score ≤ 2), interested in participating in organised physical activity, fluent in Swedish and not currently obtaining maintained HEPA levels for at least 6 months [20]. Participants were recruited by way of a mailed questionnaire [20]. Those returning the questionnaire and fulfilling the inclusion criteria were asked to participate in the intervention study. A subsample consisting of those living in the Stockholm or Umeå area was invited to participate in extra assessments of pain sensitivity and EIH in addition to performance tests and having anthropometric data collected for all study participants. Twenty persons without major joint or muscle pain (16 women and 4 men; mean age, 60 years [SD, 6]), matched for age and sex, served as HC and were recruited via advertisements posted at Karolinska Institutet and Karolinska University Hospital in Huddinge, Sweden.

### Assessments

In the present study we used baseline data from the PARA intervention study collected with questionnaires and pressure algometry.

#### Questionnaires

The questionnaires included information about the following:

1. Health-related quality of life as measured with the EuroQol-5D thermometer (EQ 5D) assessing health on the current day from ‘worst imaginable health state’ (=0) to ‘best imaginable health state’ (=100). The EQ-5D has construct validity in persons with RA, is responsive to change and is satisfactorily reliable for group comparisons [21].
2. Activity limitation as measured with the HAQ-DI [22]. The HAQ-DI comprises 20 questions addressing activities of daily living performed within the past week: dressing and grooming, arising, eating, performing personal hygiene, reaching, gripping, walking and engaging in common daily activities. Each item is scored from ‘with no difficulty’ (=0) to ‘unable to perform’ (=3) The HAQ-DI is valid and reliable in persons with RA [23].
3. HEPA as measured with the International Physical Activity Questionnaire short version, a self-administered questionnaire about physical activity at several intensity levels and across the domains of home, work, transport and leisure undertaken over the 7 days before the assessment. The short version has acceptable test-retest reliability and criterion-related validity [24].

#### Algometry

Pressure algometry [25] at rest and during standardised static muscle contractions [26] was used to assess PPTs and EIH. The algometer (Somedics Production AB, Hörby, Sweden) had a probe area of 1 cm^2^, and the pressure increase was kept at a rate of approximately 50 kPa/second [25]. Four physiotherapists trained to use the equipment performed assessments.

#### Assessments of PPTs

The assessment of PPTs included information about the following:

1. Global pain at rest was rated on a visual analogue scale (VAS; range, 0–100) before PPTs were assessed.
2. PPTs were assessed at rest bilaterally once at six sites (left and right *m. supraspinatus*, *m. gluteus maximus*, and the lateral epicondyles). The participants were instructed to indicate when the pressure sensation became painful by pressing the push button on the algometer. The mean of the stimulus intensity (kPa) of the six sites was calculated for each participant and used as the individual PPT mean.
3. Suprathreshold pressure pain sensitivity at rest was assessed at the same six sites. The participants were asked to indicate when the pressure reached an intensity rated as 4/10 and 7/10 on the Borg category ratio 10 scale [27, 28]. The means of the stimulus intensity (kPa) of the six sites (4/10 and 7/10) were calculated for each participant and used as suprathreshold 4/10 and suprathreshold 7/10.

#### Assessment of EIH

The assessment of EIH included information about the following:

1. The maximum voluntary contraction force (MVC) of the right leg extensors was determined as a basis for the assessment of EIH. MVC was tested using a Biodex Multi-Joint System 4 Pro dynamometer (Biodex Medical Systems, Shirley, NY, USA) with the participant in a sitting position with hip and knee joints flexed to 90 degrees and hands resting in the lap. Three 5-second measurements of MVC were taken with 1-minute rests in between. The highest of the three recorded values was determined to be each participant’s MVC.
2. PPTs at rest were assessed at the right *m. quadriceps* and the left *m. deltoideus* before the contraction to establish baseline values.
3. EIH assessment was based on one submaximal isometric contraction. Segmental EIH was assessed at the contracting right *m. quadriceps*, and plurisegmental EIH was assessed at the resting left *m. deltoideus*. The contraction was performed with the participants sitting in the Biodex dynamometer with their hip and knee joints flexed to 90 degrees. They were instructed to perform a right-leg isometric knee extension contraction and to maintain it until they were unable to sustain 30% of their MVC, as indicated by the Biodex dynamometer (maximum 5 minutes). Throughout the contraction PPTs were assessed twice per site (right *m. quadriceps* and left *m. deltoideus*) every 30 seconds.
4. The worst thigh pain during contraction was rated on a VAS (0–100) after the isometric muscle contraction until exhaustion.

### Statistics

Descriptive data are presented as mean and SD for parametric variables and as median and 25th–75th percentiles for non-parametric data. Differences between groups in parametric data were calculated with the independent samples test and in non-parametric data with Mann-Whitney *U* test, and the level of significance was set at 0.05.

#### Segmental and plurisegmental EIH

Because baseline PPT values can be expected to vary considerably within individuals [25] and also to differ between participants with RA and HC, the relative change in PPTs during contraction was analysed to assess EIH. As in previous studies [13, 15], the PPTs were normalised by dividing each PPT value for each participant by his or her first PPT measure at the corresponding site (the first PPT at baseline).

The values divided by the first PPT at baseline were the second PPT value of the two at baseline (nPPTbase), the first PPT during contraction (nPPTstart), the middle value or (if uneven number of PPTs) the mean value of the two middle values (nPPTmid), and the last PPT (nPPTend) for each individual at *m. quadriceps* (segmental EIH) and *m. deltoideus* (plurisegmental EIH). The mean and SD values were calculated.

The segmental EIH effects were assessed by analysing normalised PPTs at the contracting *m. quadriceps* using repeated measures analysis of variance with the within factor time (four levels: at baseline and three times during contraction [start, middle and end]) and the between-subject factor group (RA and HC). Greenhouse-Geisser corrections were used in cases of significant tests of sphericity. The plurisegmental EIH effects were analysed in the same way using the normalised PPTs of the distant resting *m. deltoideus*.

## Results

### Participant characteristics

The participants with RA did not differ significantly from HC in age or sex, but they rated their health-related quality of life lower and activity limitation higher than HC, and they were also less physically active (Table 1).

**Table 1:** Characteristics and baseline information for participants with rheumatoid arthritis and healthy control subjects

	RA (*n* = 46)	HC (*n* = 20)	*p* Value
Age, years, mean (SD)	61 (9)	60 (6)	n.s.
Female sex, *n* (%)	43 (94)	16 (80)	n.s.
Disease duration, years, median (25th–75th percentiles)	9 (5–19)	n.a.	n.a.
HRQoL (EQ-5D), 0–100, median (25th–75th percentiles)	80 (65–85)	91 (90–98)	<0.001
Activity limitation (HAQ-DI), 0–3, median (25th–75th percentiles)	0.50 (0.09–0.88)	0 (0.00–0.00)	<0.001
HEPA (IPAQ), yes/no, *n* (%)	34/12 (74/26)	19/1 (95/5)	0.049

*Abbreviations: RA* Rheumatoid arthritis, *HC* Healthy control subjects, *n.s.* Non-significant, *HRQoL* Health-related quality of life, *EQ-5D* EuroQol-5 thermometer, *HAQ-DI* Health Assessment Questionnaire Disability Index, *HEPA* Health-enhancing physical activity, *IPAQ* International Physical Activity Questionnaire, *n.a.* not assessed

### PPTs

The participants with RA rated global pain at rest higher than HC. They also had lower PPTs than HC (Table 2).

**Table 2:** Pressure pain thresholds

	RA (*n* = 45)	HC (*n* = 20)	*p* Value
Global pain at rest (VAS), 0–100, median (25th–75th percentiles)	11 (0–24)	0 (0–0)	<0.001
PPTs (kPa)			
Individual PPT mean, kPa, mean (SD)	318 (146)	487 (194)	<0.001
Suprathreshold 4/10, kPa, mean (SD)	433 (202)	638 (215)	0.001
Suprathreshold 7/10, kPa, mean (SD)	620 (285)	851 (236)	0.002

*Abbreviations: RA* Rheumatoid arthritis, *HC* Healthy control subjects, *PPT* Pressure pain threshold, *VAS* Visual analogue scale

Data are presented for global pain at rest, PPTs at rest (individual PPT mean) and suprathreshold pressure pain (suprathresholds 4/10 and 7/10) for participants with RA and HC

### EIH

Absolute PPTs at baseline were significantly lower for participants with RA than for HC, both at *m. quadriceps* (RA mean, 548 kPa [SD, 308]; HC mean, 799 kPa [SD, 291]; *p* = 0.004) and at *m. deltoideus* (RA mean, 261 kPa [SD, 130]; HC mean, 376 kPa [SD, 164]; *p* = 0.005).

#### Segmental EIH

**Fig. 1:**
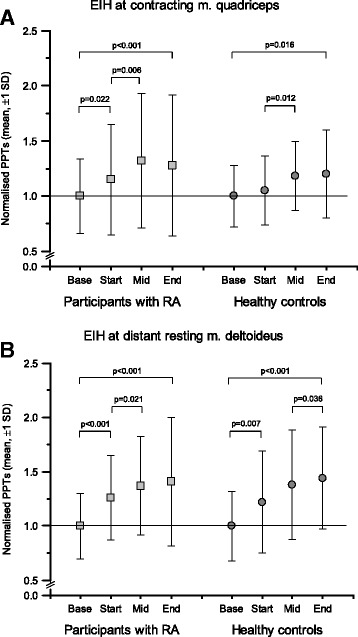
**a** and **b** Normalised pressure pain thresholds (PPTs) (mean ± SE) at the contracting (**a**) or distant resting (**b**) muscle at baseline, at the start of, in the middle of and at the end of a standardised contraction of *m. quadriceps* corresponding to 30% of maximum voluntary contraction force. Each PPT value was normalised and adjusted (by adding a coefficient) so that the baseline value always corresponds to 1. Values > 1.0 indicate higher PPTs during contraction (i.e., activation of exercise-induced hypoalgesia [EIH])

The mean time for the submaximal isometric contractions (maximum, 5 minutes) was 4 minutes and 6 seconds for participants with RA (mean MVC, 105 N) and 3 minutes and 40 seconds for HC (mean MVC, 135 N). Normalised PPTs at the contracting *m. quadriceps* increased significantly in both groups (RA, 0.99 vs 1.27; *p* < 0.001; HC, 0.89 vs 1.10; *p* = 0.016) alike (*p* = 0.531) from baseline to the end of the *m. quadriceps* contraction, indicating a functioning segmental EIH (Fig. 1a).

There was a significant effect for the factor time (*df* =2.288, *F* = 11.657, *p* < 0.001), but no significant effect was seen for group, nor did we find a significant time × group interaction. Post hoc analysis revealed a significant increase in normalised PPTs from baseline to end of contraction in both groups. Increases in normalised PPTs also occurred in participants with RA from baseline to the first PPTs during contraction, as well as in both participants with RA and HC from the first PPTs during contraction to the PPT at the middle of the contraction (Fig. 1a).

#### Plurisegmental EIH

Normalised PPTs at the distant resting *m. deltoideus* increased significantly in both groups (RA, 0.95 vs 1.36; *p* < 0.001; HC, 0.87 vs 1.31; *p* < 0.001) alike (*p* = 0.863) from baseline to the end of the quadriceps contraction, indicating a functioning plurisegmental EIH (Fig. 1b). There was a significant effect for the factor time (*df* = 2.196, *F* = 26.182, *p* < 0.001), but no significant effect was seen for group, nor did we find a significant time × group interaction. Post hoc analysis revealed a significant increase in normalised PPTs from baseline to end of contraction in both groups. Increases also occurred in both participants with RA and HC from baseline to the first PPTs during contraction and from the first PPTs during contraction to the PPT at the middle of the contraction (Fig. 1b). The worst thigh pain perceived during contraction was rated higher (*p* = 0.003) by the participants with RA than by HC (RA median, 22; 25th–75th percentiles, 2–52, HC median, 0; 25th–75th percentiles, 0–26).

## Discussion

To our knowledge, this is the first study exploring pressure pain sensitivity and activation of EIH in a sample of both men and women with RA and comparing them with HC without major pain. Our main findings indicate that persons with RA have higher pain sensitivity but a normal activation of segmental and plurisegmental EIH. Our results thus confirm those derived from our previous pilot study [15] and offer a possible explanation for pain reduction observed in persons with RA following clinical exercise programmes.

Higher pressure pain sensitivity at muscle tissue in persons with RA at rest as found in the present study suggests involvement of central pain mechanisms (e.g., central sensitisation, including facilitation and/or disinhibition) in RA pain. These findings agree with results of previous studies [5, 16, 29] in which researchers reported decreased pressure pain thresholds at non-inflamed tissue in patients with RA. The present results also confirm the findings of our previous pilot study [16] demonstrating hypersensitivity in postmenopausal women with RA and further support that persons with RA may have dysfunctional pain processing with widespread central sensitisation.

Our findings of higher ratings of global pain at rest and in worst pain in the thigh during contraction among participants with RA compared with HC indicate that persons with RA may experience increased pain during exercise. However, the pain ratings represented a wide range, especially those of ‘worst pain during exercise’. This might be imperative to individualise exercise support, such as in the context of the fear-avoidance model, where pain intensity is suggested to predict disability mediated by fear avoidance [30]. In our recent study, high fear avoidance was correlated with male sex, low income, high pain ratings, poor perceived health, low health-related quality of life and low exercise self-efficacy [31]. Thus, pain is a complex phenomenon, and numerous factors other than pain physiology need to be taken into account when tailoring therapeutic exercise to an individual person with RA.

We found normal activation of pain-inhibitory mechanisms during static contraction, both segmental and plurisegmental, in the participants with RA. Another study exploring endogenous pain modulation in response to submaximal exercise on a bicycle ergometer in RA [14] found a normal decrease in temporal summation of painful stimuli in patients with RA, thus suggesting a decrease in pain-facilitatory mechanisms during dynamic exercise in RA.

In other chronic pain conditions, different responses in pain modulation have been found during exercise. In a previous study assessing patients with hip or knee OA, we found increased pressure pain sensitivity, but a normal function of segmental and plurisegmental EIH, during static contractions [25], which is in accordance with our present findings in persons with RA. In contrast, patients with whiplash-associated disorders were found to have increased temporal summation of pain, suggesting dysfunction in pain-inhibitory mechanisms [32]. Furthermore, patients with FM showed hyperalgesia and no activation of pain-inhibitory mechanisms, segmental or plurisegmental, during static muscle contractions. Rather, an increase in pain sensitivity was seen [10, 13]. Patients with shoulder myalgia showed an activation of the pain-inhibitory mechanisms when contracting a non-painful muscle distant to the pain but a lack of activation when contracting the painful muscle [13]. Thus, on the basis of earlier studies, EIH seems to be normally activated when exercising non-painful but not painful muscles, as well as normally activated in patients with chronic joint pain such as in OA.

In populations with RA, FM is a quite common co-morbidity [33, 34]. One hypothesis presented is that the dysfunction in pain processing in RA has similarities to the dysfunction found in FM [33]. Our results indicate that the dysfunction in pain processing differs between RA and FM; the participants with RA showed augmented pain sensitivity similar to FM but normal activation of the pain-inhibitory mechanisms during muscle contraction. Interestingly enough, the same pattern has been observed in OA [15]. With these results in mind, further exploration of pain processing during a long-term physical activity programme would add important information about the nature of the pain-processing dysfunction in RA.

One strength of our study is its sample, which has to be considered suitable for one including standardised assessments by algometry of PPTs as well as EIH. One limitation is that no disease activity data were available for our study participants. However, our recordings of pain, health-related quality of life and activity limitation, taken together, indicate that disease activity was low to moderate. Another possible limitation is selection bias, with the inclusion of participants with RA being limited to those interested and fit enough to participate in a physical activity support programme. For example, persons with HAQ-DI values > 2 would, by definition, not be able to exercise independently. Thus, our results can be generalised only to populations with RA that are most likely to have a chance to influence their pain sensitivity with exercise. The fairly high dropout rate due to incomplete data was the result of an administrative error and should not influence the external validity of our results. This was also confirmed by the dropout analysis indicating no major differences between those with complete data and those with incomplete data.

Despite our study’s indication that persons with RA might be able to reduce their pain with exercise, the optimal type and dose for adequate pain reduction need to be further explored. Although controlled trials in clinical or research settings are the most frequent study design for evaluating pain reduction by exercise, future studies should also explore the potential effects on pain sensitivity and EIH of physical activity outside health-care contexts.

## Conclusions

Our results indicate a generally increased pain sensitivity but normal function of EIH among persons with RA and thus offer one possible explanation for pain reduction observed in this group of patients following clinical exercise programmes. More research is needed to explore the adequate exercise type and dose for optimal response in terms of pain modulation.

## Abbreviations

EIH: Exercise-induced hypoalgesia
EQ-5D: EuroQol-5D thermometer
FM: Fibromyalgia
HAQ-DI: Health Assessment Questionnaire Disability Index
HC: Healthy control subjects
HEPA: Health-enhancing physical activity
HRQoL: Health-related quality of life
IPAQ: International Physical Activity Questionnaire
MVC: Maximal voluntary contraction
n.s.: Not significant
nPPTbase: Second pressure pain threshold value of two at baseline
nPPTend: Last pressure pain threshold value
nPPTmid: Middle or mean value of two middle pressure pain threshold values
nPPTstart: First pressure pain threshold during contraction
OA: Osteoarthritis
PARA: Physical Activity in Rheumatoid Arthritis 2010 study
PPT: Pressure pain threshold
RA: Rheumatoid arthritis
VAS: Visual analogue scale
